# A Case With IgG4-Related Spinal Pachymeningitis Causing Spinal Cord Compression

**DOI:** 10.3389/fneur.2020.00500

**Published:** 2020-07-14

**Authors:** Rensheng Zhang, Jiguo Gao, Teng Zhao, Beilin Zhang, Chenglin Wang, Chao Wang, Lexiang Cui, Jiafeng Chen, Shaokuan Fang

**Affiliations:** Department of Neurology, Neuroscience Center, The First Teaching Hospital of Jilin University, Changchun, China

**Keywords:** immunoglobulin G4 (IgG4)-related disease, spinal pachymeningitis, spinal cord compression, case report, systemic disease, histopathology and immunohistochemistry

## Abstract

Immunoglobulin G4 (IgG4)-related disease is a systemic disease characterized by sclerosing lesions and an increased serum IgG4 level. This condition can involve any organ, but IgG4-related spinal pachymeningitis is relatively rare. In the current study, we report a case of spinal cord compression caused by IgG4-related spinal pachymeningitis. A 39-year-old man presented to us with a 15-day history of back pain and a 3-day history of dysuresia, exacerbated by weakness in the lower extremities for 2 days. Cervical magnetic resonance imaging (MRI) showed strip-shaped abnormal signals along the anterior and posterior borders of the spinal cord at the C5–T4 levels. The IgG level in cerebrospinal fluid was 718.0 mg/L. Thoracic MRI revealed strip-shaped abnormal signals with remarkable enhancement along the anterior and posterior borders of the dural sac at the T1–T6 levels. Histopathological examination confirmed IgG4-related spinal pachymeningitis. The symptoms worsened rapidly, and surgical resection of the space-occupying lesion in the vertebral canal was performed for spinal decompression. Corticosteroid therapy was administered, and the patient's motor functions were mildly improved. IgG4-related disease can manifest as spinal pachymeningitis and cause spinal cord compression. Clinicians should be aware of this rare condition, and early diagnosis, timely surgical decompression, and appropriate corticosteroid therapy should be highlighted.

## Introduction

Immunoglobulin G4 (IgG4)-related disease is a systemic disease characterized by sclerosing lesions and an elevated serum IgG4 level. This disease can involve virtually any organ, and pancreas, salivary glands, and lacrimal glands are most commonly affected. In addition, IgG4-related central nervous system (CNS) involvement is a rare and distinct entity of IgG4-related disease (1), with hypertrophic pachymeningitis and hypophysitis being the most common manifestations (2). It can also involve parenchyma, peri-ventricular area, and cranial nerves (2). However, IgG4-related disease manifesting as spinal pachymeningitis, which may represent a distinct subtype of CNS involvement, is relatively rare (3–5). Due to the rarity of identified cases, the clinical and radiological characteristics of IgG4-related spinal pachymeningitis have yet to be well-elucidated, and this disease is usually misdiagnosed. Spinal cord compression refers to a condition in which the spinal cord is compressed by a space-occupying lesion in the vertebral canal; tumor and inflammatory lesions are the most common contributors (6). In the current study, we report a case of spinal cord compression caused by IgG4-related spinal pachymeningitis. Additionally, the relevant literature was reviewed.

## Case Presentation

A 39-year-old previously healthy man presented to us with a 15-day history of back pain and a 3-day history of dysuresia, exacerbated by weakness in the lower extremities for 2 days. One day prior to admission, he was unable to walk or urinate. He denied any history of hypertension, diabetes, heart diseases, infectious diseases, or trauma as well as any history of direct contact with cattle or sheep or tuberculosis. On admission, physical examination showed a loss of sensation below the T12 dermatome, spinal tenderness at the T2 level, and muscle strength of grade 3/5 in the bilateral lower extremities. Additionally, there was radicular pain during cervical anteflexion. The muscular tone was normal in all extremities. The proprioceptive sensation in the lower extremities was also decreased, and the abdominal and cremasteric reflexes disappeared. Ankle clonus, Hoffmann sign, Babinski sign, and Chaddock sign were positive for bilateral extremities. On the second day after admission, the lower extremity weakness was exacerbated. Neurological reexamination showed a loss of sensation below the T8 dermatome and a muscle strength of grade 2/5 in the bilateral lower extremities; nevertheless, the bilateral pathological reflexes (Hoffmann sign, Babinski sign, and Chaddock sign) were all negative.

**Figure 1:**
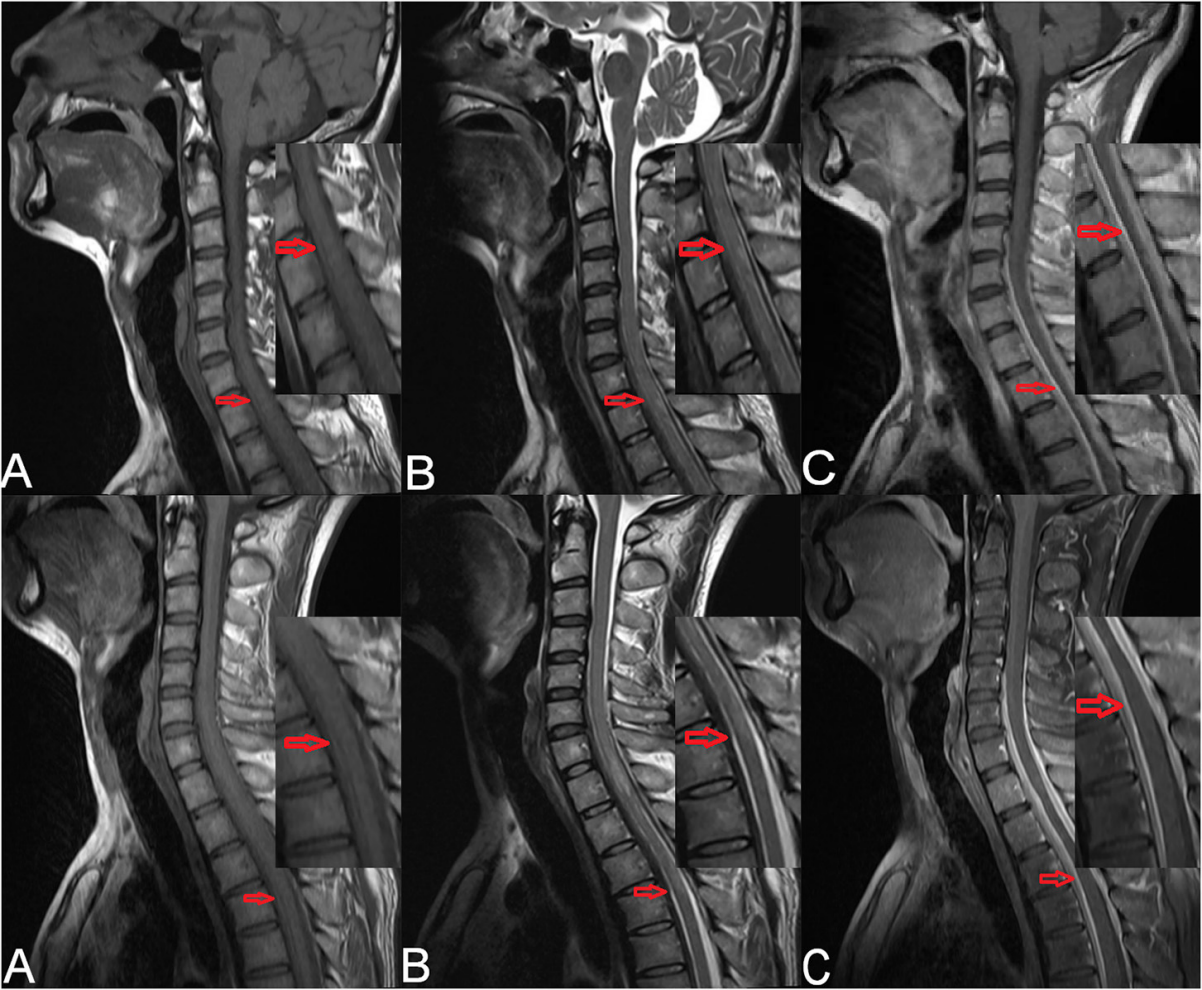
Preoperative cervical MRI of the patient (upper). **(A)** Sagittal T1-weighted imaging showed strip-shaped hypointensity along the anterior and posterior borders of the spinal cord at the C5–T4 levels. **(B)** Sagittal T2-weighted imaging showed strip-shaped hyperintensity along the anterior and posterior borders of the spinal cord at the C5–T4 levels. **(C)** Contrast-enhanced T1-weighted imaging showed linear enhancement at the C5–T4 levels (red arrows). Cervicothoracic MRI of the patient after symptom aggravation (lower). **(A)** Sagittal T1-weighted imaging showed strip-shaped hypointensity along the anterior and posterior borders of the dural sac at the T1–T6 levels. **(B)** Sagittal T2-weighted imaging showed strip-shaped hyperintensity along the anterior and posterior borders of the dural sac at the T1–T6 levels. **(C)** Contrast-enhanced T1-weighted imaging showed linear enhancement along the anterior and posterior borders of the dural sac at the T1–T6 levels (red arrows).

Cervical magnetic resonance imaging (MRI) showed strip-shaped abnormal signals along the anterior and posterior borders of the spinal cord at the C5–T4 levels (Figure 1, upper). Cerebrospinal fluid (CSF) examination via a lumbar puncture showed faint-yellow fluid with a normal pressure of 130 mmH_2_O. The protein level was elevated (2.7 g/L), and Pandy's test showed strong positivity. Cell counting showed elevated leukocytes (31 × 10^6^/L) and erythrocytes (400 × 10^6^/L) with a lymphocyte ratio of 98% and a monocyte ratio of 7%. The IgG level in the CSF was 718.0 mg/L. The CSF smear and IgG antibody for tuberculosis were negative, and quantitative examination and agglutination test for brucellosis were negative as well.

After admission, methylprednisolone (1,000 mg) was administered intravenously. Considering that the subdural extramedullary lesion might be caused by a hematoma or tuberculosis, methylprednisolone was used for 2 days and then replaced by an antituberculosis therapy. Five days later, the symptoms became aggravated. Cervicothoracic MRI revealed strip-shaped abnormal signals along the anterior and posterior borders of the dural sac at the T1–T6 levels (Figure 1, lower). Physical examination showed a muscle strength of grade 0/5 in the bilateral lower extremities. A diagnosis of spinal cord compression was made, and surgical resection of the space-occupying lesion was scheduled.

Intraoperatively, the lesion showed a rubber-like appearance with a hard nature, and it was distributed extensively on the dura mater. The dura mater had no pulsation with a high tension. After the dura mater was incised, a subdural lesion was noted with a rubber-like appearance as well, and the spinal cord was compressed. The subdural and epidural lesions were resected for decompression. The post-operative course was uneventful. Physical examination showed a loss of sensation below the T4 dermatome, and muscle strength of grade 0/5 in the bilateral lower extremities. The bilateral pathological reflexes (Babinski sign and Chaddock sign) were all positive.

**Figure 2:**
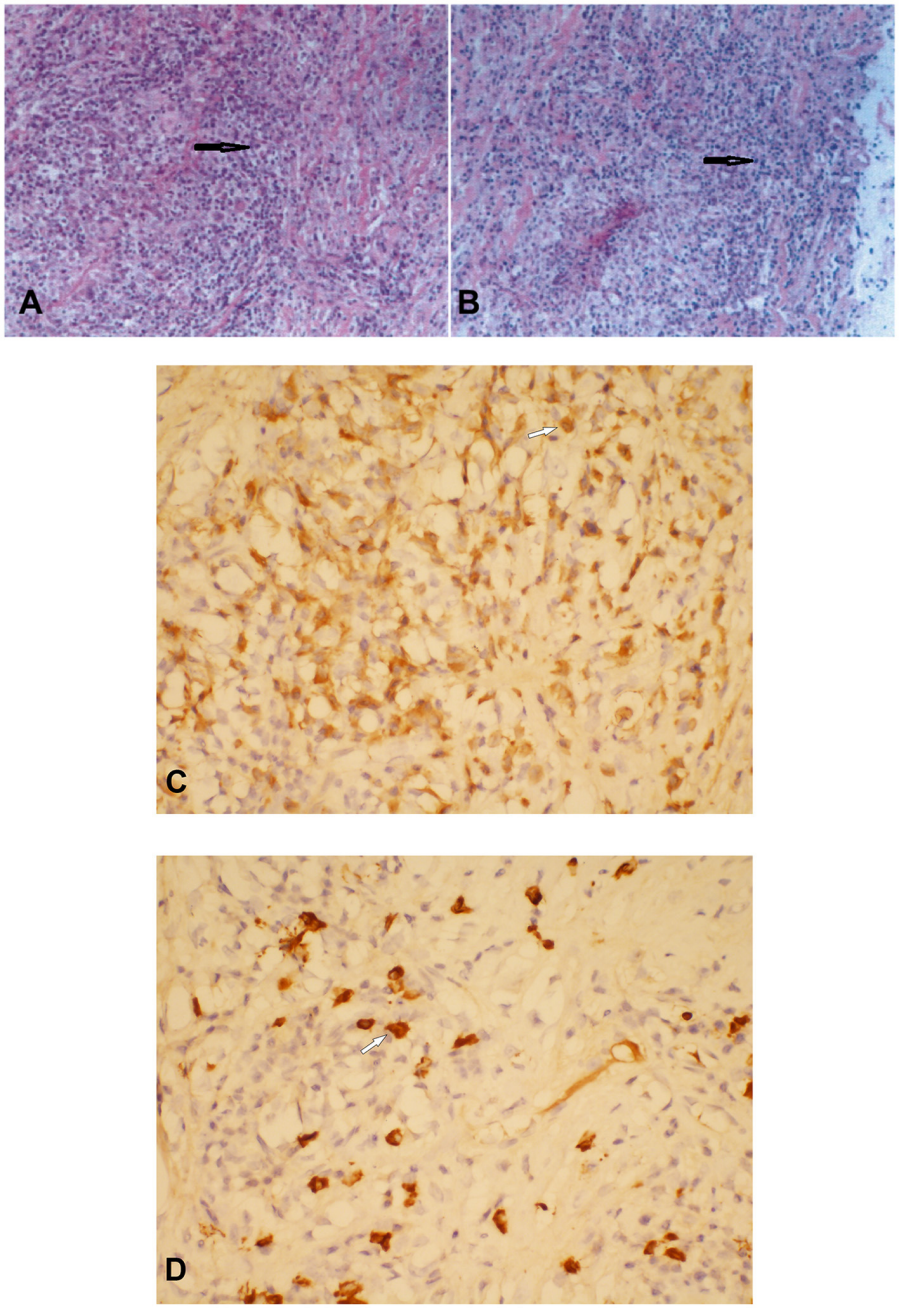
Histopathological and immunostaining examination of the thoracic subdural lesion which was resected. Hematoxylin–eosin staining showed numerous plasma cell infiltrates within the fibrofatty tissue (**A** × 200; **B** × 100) (black arrows). Plasma cells were positive for immunoglobulin G (IgG) **(C)** and IgG4 **(D)** (**C** × 400; **D** × 400) (white arrows).

Histopathological examination showed diffuse infiltration of inflammatory cells within the fibrofatty tissue, and numerous plasma cell infiltrates could be noted (Figures 2A,B). Immunohistochemical staining showed that the plasma cells were positive for IgG and IgG4. Focally, more than 10 IgG4-positive plasma cells could be seen per high-power field (HPF) (Figures 2C,D). Additionally, perivascular histiocytosis was arranged in clusters with scattered multinuclear giant cells, which were positive for CD68 but negative for CD1α. A diagnosis of IgG4-related sclerosing disease with histiocytosis was made. The Ki-67 labeling index reached 30%.

Further laboratory examination revealed an IgG4 level of 1.050 g/L, an erythrocyte sedimentation rate of 120 mm/1 h, and a C-reactive protein level of 115 mg/L. Based on the clinical and histopathological profiles, a diagnosis of spinal cord compression caused by IgG4-related spinal pachymeningitis was made. Corticosteroid therapy was scheduled (Table 1). Two days following the corticosteroid administration, the muscle strength in the right lower extremity returned to grade 1/5. Methylprednisolone was used for 3 days before the patient was discharged, and he was told to continue small-dose methylprednisolone maintenance therapy. When the patient was discharged, the muscle strength in the left lower extremity had returned to grade 1/5 and that in the right lower extremity had returned to grade 2/5; the sensory functions remained unchanged, and bilateral pathological reflexes (ankle clonus, Hoffmann sign, Babinski sign, and Chaddock sign) remained positive. During the subsequent follow-up period, the patient's condition was stable and the corticosteroid was decreased 4 mg per week.

**Table 1:** Treatment and laboratory parameters.

**Dosage of methylprednisolone (mg)**	**Duration of treatment (days)**	**Erythrocyte sedimentation rate (mm/1 h)**	**C-reactive protein (g/L)**
1,000	3	99	40
500	3	54	8.49
240	6	28	5.62
120	3	44	9.29

## Discussion

The identification of IgG4-related disease can be traced back to 1961, when Sarles et al. (7) reported that the pathogenesis of chronic pancreatitis may be related to autoimmunity. In 1995, Yoshida et al. (8) originally proposed the term “*autoimmune pancreatitis*,” and then in 2001, autoimmune pancreatitis was found to be related to IgG4-positive plasma cells (9). In 2003, Kamisawa et al. (10) first proposed the concept of “*IgG4-related autoimmune disease*.” In 2010, Takahashi et al. (11) formally named this syndrome “*IgG4-related disease*.” Japanese scholars have reported an incidence of 2.8–10.8 per million (12–14). However, until now, no large-scale epidemiological data have been made available worldwide.

IgG4-related diseases usually affect elderly patients (>50 years), with a significant male predominance (11, 15). Clinical manifestations of IgG4-related diseases include (1) involvement of multiple organs, (2) fibroplasia or sclerosis in single or multiple organs, (3) extensive infiltration of IgG4-positive lymphocytes and plasma cells, (4) elevated serum IgG4 level (≥1,350 mg/L), and (5) response to glucocorticoids (11, 15–17). There has been no consensus regarding the clinical diagnostic criteria of IgG4-related diseases. In 2011, Okazaki et al. (13) proposed the criteria as follows: (1) diffuse or localized swelling/lumps in single or multiple organs; (2) serum IgG4 ≥1,350 mg/L; (3) histopathological findings: A. remarkable infiltration of lymphocytes and plasma cells and fibrosis; B. infiltration of IgG4-positive plasma cells (>10 IgG4-positive plasma cells/HPF); C. storiform fibrosis; and/or D. obliterative phlebitis. A diagnosis of IgG4-related disease can be made based on (1) + (2), (1) + (3) A and B, (2) + (3) A and B, or (3) A–D (18). The current case met the diagnostic criteria (1) + (3) A and B.

Although the majority of IgG4-related diseases are characterized by elevated serum levels of IgG and IgG4, according to the literature, IgG and IgG4 can also remain within the normal ranges in 30 and 8% of all patients, respectively (18, 19). Thus, serum IgG and IgG4 elevation cannot be used as a specific marker for the diagnosis of IgG4-related diseases. There may also be no specific findings in routine CSF tests. Besides, the meningeal biopsy is usually required for final diagnosis, but it is not easy to implement in fact due to the risks and technical difficulties. Since IgG4 production is a key pathological feature of IgG4-related diseases, the analysis of CSF for intrathecal IgG4 production and IgG4 Index as a useful, non-invasive, and cost-affordable tool may be a choice for the diagnosis of IgG4-related diseases (20).

**Figure 3:**
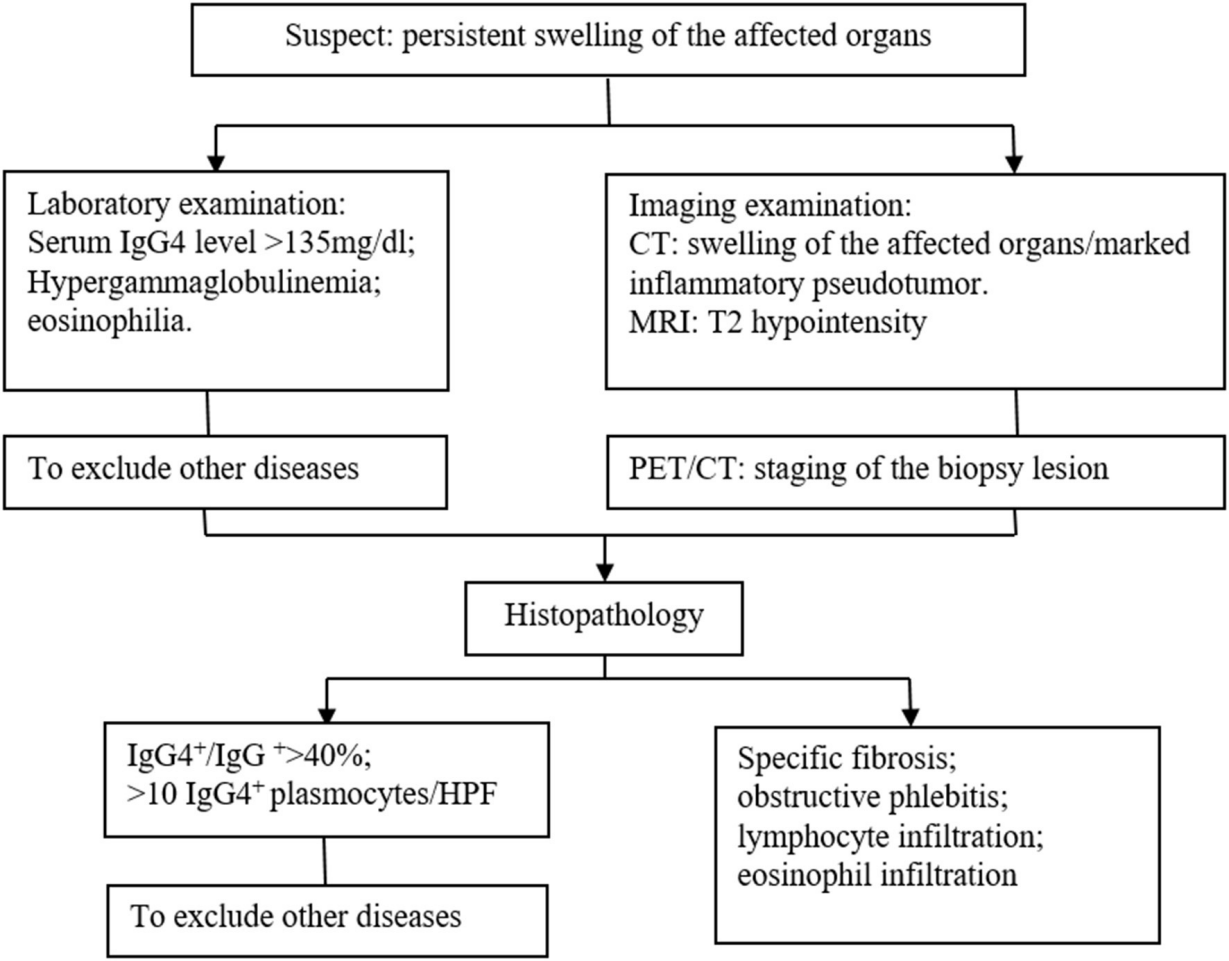
Diagnosis flow of immunoglobulin G4 (IgG4)-related disease.

The identification of IgG4-related diseases should depend on comprehensive evaluation including clinical manifestations, laboratory examinations, radiological features, and histopathological findings, and the gold standard is histopathology and immunohistochemistry. We summarized a diagnosis flow as shown in Figure 3. In the current case, massive infiltration of plasma cells and >10 IgG4-positive plasma cells/HPF supported the diagnosis of IgG4-related disease.

Cervicothoracic spinal cord compression caused by IgG4-related disease is extremely rare (13). As the clinical manifestations and radiological features of IgG4-related disease are non-specific, early diagnosis is challenging. In the current case, the patient developed severe sensorimotor deficiencies, requiring surgical decompression. The main differential diagnoses include Wegener granulomatosis and lymphoma. Wegener granulomatosis also involves multiple organs and manifests as chronic vasculitis and infiltration of IgG4-positive plasma cells. However, Wegener granulomatosis is a special, severe form of vasculitis, mainly affecting the upper respiratory tract, lung, and kidney. In Wegener granulomatosis, the serum anti-neutrophil cytoplasmic antibody (ANCA) is usually positive, and histopathological examination reveals leukocytoclastic vasculitis with necrotic changes and granulomatous inflammation, which can aid in the differential diagnosis (21). Lymphomas with abundant lymphocytes and plasmocytes, such as marginal zone B-cell lymphomas, low-grade follicular lymphomas, and extramedullary plasmacytomas, should also be differentiated from IgG4-related diseases, and immunohistochemical CD20 positivity and restricted expression of κ/λ light chain can help the diagnosis.

IgG4-related pachymeningitis is rare and was first reported in 2009 (22). As one of the main causes of meningeal inflammatory disease, it is mainly characterized by the lack of extra-neurologic organ involvement and systemic signs (23). Histopathologic examination should be carried out if possible, as it is essential for final diagnosis because serum markers are rarely informative (23).

All the symptomatic and active IgG4-related diseases need timely treatment. For the primary treatment, glucocorticoids are the first choice for inducing remission. Moreover, for patients who respond poorly to glucocorticoids and those who experience recurrence, addition of immunosuppressors can be attempted. Currently, there is no specific treatment for IgG4-related pachymeningitis. Short-term efficacy of surgery in single-organ IgG4-related pachymeningitis is good and, in some cases, may be sufficient to achieve remission (23). When multiple organs are involved, steroid hormones are still the first choice, although some studies have shown the effectiveness of immunosuppressants such as systemic rituximab or cyclophosphamide infusions (24). However, as for the patients with organ-threatening manifestations of IgG4-related diseases due to meningeal involvement poorly responsive to systemic immunosuppressive therapies, the intrathecal rituximab might represent a valid and safe therapeutic approach (25).

In conclusion, the clinical presentations and radiological characteristics of IgG4-related CNS diseases are non-specific. The definitive diagnosis should depend on histopathology and immunohistochemistry. Spinal cord compression caused by IgG4-related disease is extremely rare and may progress rapidly, leading to severe neurological deficits. Timely surgical decompression with subsequent systemic corticosteroid treatment is needed.

## Data Availability Statement

The datasets generated for this study can be found within the article.

## Ethics Statement

This study was approved by the ethics committee of the First Hospital of Jilin University, Changchun, China. Written informed consent was obtained from the patient for the publication of this case report.

## Author Contributions

RZ, JG, and TZ contributed to the conception and design of the study. BZ and LC organized the database. CheW and ChaW performed the statistical analysis and wrote the first draft of the manuscript. JG, JC, TZ, and RZ wrote sections of the manuscript. All authors contributed to manuscript revision, read, and approved the submitted version.

## Conflict of Interest

The authors declare that the research was conducted in the absence of any commercial or financial relationships that could be construed as a potential conflict of interest.

## Abbreviations

IgG4: immunoglobulin G4
CNS: central nervous system
MRI: magnetic resonance imaging
CSF: cerebrospinal fluid
HPF: high-power field
ANCA: anti-neutrophil cytoplasmic antibody.
